# Data Recorded in Real Life Support the Safety of Nattokinase in Patients with Vascular Diseases

**DOI:** 10.3390/nu13062031

**Published:** 2021-06-13

**Authors:** Giuseppe Gallelli, Giulio Di Mizio, Caterina Palleria, Antonio Siniscalchi, Paolo Rubino, Lucia Muraca, Erika Cione, Monica Salerno, Giovambattista De Sarro, Luca Gallelli

**Affiliations:** 1 Department of Vascular Surgery, Pugliese Ciaccio Hospital, 88100 Catanzaro, Italy; giuseppegallelli@hotmail.it (G.G.); paolo.rubino@libero.it (P.R.); 2 Forensic Medicine, Department of Law, Magna Graecia University of Catanzaro, 88100 Catanzaro, Italy; 3 Department of Health Science, School of Medicine, University of Catanzaro, Clinical Pharmacology and Pharmacovigilance Unit, Mater Domini University Hospital, 88100 Catanzaro, Italy; palleria@unicz.it (C.P.); desarro@unicz.it (G.D.S.); gallelli@unicz.it (L.G.); 4 Department of Neurology and Stroke Unit, Annunziata Hospital of Cosenza, 87100 Cosenza, Italy; anto.siniscalchi@libero.it; 5 Department of General Medicine, ASP 7 Catanzaro, 88100 Catanzaro, Italy; luciamuraca@alice.it; 6 Department of Pharmacy Helath and Nutritional Sciences, Department of Excellence 2018-2022, University of Calabria, 87036 Rende, Italy; erika.cione@unical.it; 7 Department of Medical, Surgical and Advanced Technologies “G.F. Ingrassia”, University of Catania, 95041 Catania, Italy; monica.salerno@unict.it; 8 Department of Health Science, School of Medicine, Clinical Pharmacology Research Center FAS@UMG, University of Catanzaro, 88100 Catanzaro, Italy

**Keywords:** nattokinase, vascular diseases, clinical trial, low-molecular-weight heparin, Clinical Risk Management, safety of care in pharmacological treatments

## Abstract

Nattokinase (NK) is a serine protease enzyme with fibrinolytic activity. Even if it could be used for the treatment of several diseases, no data have been published supporting its use patients who underwent vascular surgery. In this study, we evaluated both the efficacy and the safety of nattokinase (100 mg/day per os) in patients admitted to vascular surgery. Patients were of both sexes, >18 years of age, with vascular diseases (i.e., deep vein thrombosis, superficial vein thrombosis, venous insufficiency), and naïve to specific pharmacological treatments (anticoagulants or anti-platelets). Patients were divided into three groups. Group 1: patients with deep vein thrombosis, treated with fondaparinux plus nattokinase. Group 2: patients with phlebitis, treated with enoxaparin plus nattokinase. Group 3: patients with venous insufficiency after classical surgery, treated with nattokinase one day later. During the study, we enrolled 153 patients (age 22–92 years), 92 females (60.1%) and 61 males (39.9%;), and documented that nattokinase was able to improve the clinical symptoms (*p* < 0.01) without the development of adverse drug reactions or drug interactions. Among the enrolled patients, during follow-up, we did not record new cases of vascular diseases. Attention to patients’ clinical evolution, monitoring of the INR, and timely and frequent adjustment of dosages represent the cornerstones of the safety of care for patients administered fibrinolytic drugs as a single treatment or in pharmacological combination. Therefore, we can conclude that the use of nattokinase represents an efficient and safe treatment able to both prevent and treat patients with vascular diseases.

## 1. Introduction

Nattokinase (NK) is a serine protease enzyme discovered in 1980 and extracted from natto, a traditional Japanese food, made by boiling or steaming soybeans and fermenting them with the *Bacillus subtilis* natto [1].

The fibrinolytic activity of nattokinase is related to a complex mechanism of action: the enzyme [1] breaks down blood clots by directly hydrolyzing fibrin and plasmin; [2] converts endogenous pro-urokinase to urokinase; [3] degrades plasminogen activator inhibitor-1, and [4] increases tissue plasminogen activator [1,2,3]. In a recent experimental study, Wu et al., [4] documented that nattokinase has anti-inflammatory and anti-oxidative stress effects, suggesting a role in the management of thrombosis.

The effects of nattokinase on the cardiovascular system have been reported in both clinical and experimental studies. Its anti-hypertensive [5,6], anti-atherosclerotic [5,7], anti-platelet/anticoagulant [8], and neuroprotective effects [9,10] were extensively documented. With the biotechnological advent NK was studied for its sensorial, pharmacokinetic and toxicology aspects as well [11,12,13,14]. These pharmacologic actions of NK have relevance to the prevention and treatment of CVD.

Nattokinase has high gastrointestinal stability (also concerning changes in pH and temperature) and presents a discrete absorption in the intestinal tract [4,5]. In a pilot clinical study, Ero et al., [6] documented that a single daily dose of nattokinase (100 mg per os) produced a peak serum level at 13.3 h ± 2.5 h with a time-dependent effect.

A fundamental feature for nattokinase is that it is used in several clinical manifestations in many other vascular diseases (i.e., atherosclerosis) without causing the development of adverse drug reactions or drug interactions [7].

However, to date, no data have been published supporting the use of NK in vascular surgery as sequential therapy. In this regard, in the present study, we evaluated both efficacy and safety of nattokinase (100 mg/days) as sequential therapy in patients admitted to vascular surgery.

## 2. Methods

### 2.1. Study Subjects

We performed an observational open-label clinical trial on patients referred to the Vascular Surgery Unit of the “Pugliese Ciaccio” Hospital of Catanzaro, from 1 April 2019 to 1 April 2020. The Local Ethics Committee approved our study protocol (120/May 2018). It was conducted in compliance with the Institutional Review Board/Human Subjects Research Committee requirements and according to the Declaration of Helsinki and the Guidelines for Good Clinical Practice criteria. Before the beginning of the study, all patients signed an informed consent.

### 2.2. Inclusion and Exclusion Criteria

Inclusion criteria: patients of both sexes, >18 years of age, with vascular diseases (i.e., deep vein thrombosis, superficial vein thrombosis, venous insufficiency), and naïve to specific pharmacological treatments (anticoagulants or anti-platelets) that signed the informed consent.

Exclusion criteria: patients < 18 years of age, with cancer and severe blood disorders, bleeding, who did not sign the informed consent or used drugs or nutrients with anticoagulant or anti-platelet activity.

### 2.3. Experimental Protocol

After clinical, laboratory, and instrumental evaluation, the admitted patients were enrolled into three groups:

**Group 1:** patients with deep vein thrombosis, treated with fondaparinux (7.5 mg/day subcutaneous) for 30 days (T1) and then nattokinase (tablet, 100 mg/2000 FU/daily per oral administration for 30 days) (T2).

**Group 2:** patients with superficial vein thrombosis or phlebitis, treated with enoxaparin (6000 IU/day subcutaneous, for 30 days) (T1). Nattokinase (100 mg/2000 FU/daily per oral administration for 30 days) (T2). 

**Group 3:** patients who underwent classical surgery (ligature and section at the saphenofemoral junction and collateral veins, with saphenousectomy). Patients suffered from venous insufficiency (T1). After surgery, nattokinase (100 mg/2000 FU/daily per oral administration for 30 days) was started (T2).

In agreement with international guidelines [15], during follow-up (every month for 6 months), the visual analogue scale (VAS) was used for pain evaluation and a Doppler ultrasonography study was performed as well as blood evaluation of ERS. Moreover, the presence of edema was evaluated in agreement with the method of Brodovicz et al. [16], where Grade 0 indicate the absence of edema, and grade 4 the presence of a severe edema (very deep pit, 8 mm).

Furthermore, in agreement with our previous studies [17,18,19,20,21], the Naranjo scale and the drug-interaction probability scale were used to evaluate the safety of the compound and the drug interactions, respectively.

### 2.4. Endpoints

The first endpoint was the statistically significant improvement (*p* < 0.05) of clinical symptoms in patients 30 days after the beginning of nattokinase administration (100 mg/day) (T2), for (T1). The second endpoint was the absence of adverse drug reactions or drug interactions in patients treated with nattokinase recorded at the end of the study concerning the admission and follow-ups.

### 2.5. Sample Size and Statistical Analysis

The primary outcome for the power calculation was the improvement of clinical symptoms 30 days after nattokinase treatment. In agreement with previously published studies [22,23], to detect a clinically relevant difference between each group, 40 subjects for each group were needed (power > 85%, alpha 0.05, two-tailed).

All data were analyzed using the IBM SPSS21 statistical program (Microsoft, Redmond, WA, USA). Mean and standard deviation (SD) were taken into consideration for the analysis. For normality, data parameters were evaluated using the Shapiro–Wilk normality test. The Anova test was used to evaluate the difference in each group, while the Student’s *t*-test was used to evaluate the difference between groups. The values are expressed as mean ± SD, and the differences were considered significant for *p* < 0.05.

## 3. Results

### 3.1. Population

During the study, we enrolled 153 patients (age 22–92 years, mean 56.3 ± 15.9 years), 92 females (60.1%; age 33–92 years, mean 57.9 ± 16.1 years) and 61 males (39.9%; age 22–81 years; mean 55.8 ± 15.3 years), without difference between the groups (*p* = 0.632, mean −1.9 ± 17.9, CI 95% −9.1, 5.2).

After clinical evaluation, the enrolled patients were divided in three groups:

**Group 1:** 50 patients (32 females and 18 males), age 32–78 years (mean 55.2 ± 14.2 years) without differences by gender (females: mean 55.8 ± 13.5 years; males: 53.8 ± 14.2 years; *p* = 0.91).

**Group 2:** 57 patients (34 females and 23 males), age 22–92 years (mean 60.7 ± 19.7 years) without differences by gender (females: mean 62.3 ± 20.5 years; males: 58.3 ± 18.6 years; *p* = 0.86) or with respect to body mass index, drugs used, or co-morbidity. 

**Group 3:** 46 patients (26 females and 20 males), age 42–67 years (mean 56 ± 7.8 years), without differences by gender (females: mean 51.7 ± 5.9 years; males: 53 ± 14.1 years; *p* = 0.9) (Table 1).

### 3.2. Effects of Drug Treatment

All patients enrolled in Group 1 were treated with fondaparinux (7.5 mg/day) for 30 days (T1) and presented a significant decrease in clinical symptoms (VAS spontaneous from 8 to 5; VAS after digital pressure from 9 to 5, *p* < 0.01) and an improvement in vascular echo-color Doppler analysis. The 30-day treatment with nattokinase induced complete remission of the clinical symptoms (*p* < 0.01).

**Figure 1:**
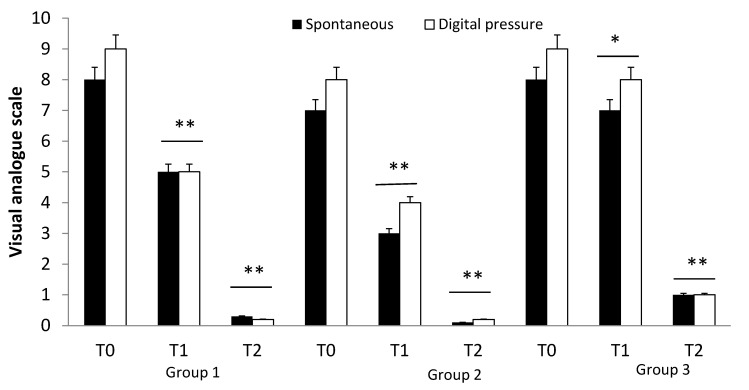
Effect of drug treatment, at different times (T0: admission), in patients enrolled in three groups. Group 1: patients treated with fondaparinux (7.5 mg/day subcutaneous) for 30 days (T1) and then nattokinase (for 30 days, T2). Group 2: patients treated with enoxaparin (6000 IU/day s.c.) for 30 days (T1) and the nattokinase (for 30 days) (T2). Group 3: Patients underwent classical surgery including ligature and section at the saphenofemoral junction and collateral veins and saphenousectomy (T1) and were treated one day later with nattokinase (for 30 days). * *p* < 0.05; ** *p* < 0.01.

In Group 2, patients were treated with enoxaparin (6000 IU/day for 30 days) and showed a significant decrease in the clinical symptoms (VAS spontaneous from 7 to 3; VAS after digital pressure from 8 to 4, *p* < 0.01) and an improvement in vascular echo-color Doppler analysis. In these patients, nattokinase was started after the discontinuation of enoxaparin. In this group, there was a complete remission of the clinical symptoms (*p* < 0.01) (Figure 1).

Finally, in Group 3, nattokinase (100 mg/day for 30 days) was started one day after vascular surgery and induced complete remission of the symptoms in all treated patients (*p* < 0.01) (Figure 1).

In patients enrolled in these groups, we did not record the development of ADRs or drug interactions.

## 4. Discussion

In this study, we evaluated the efficacy of nattokinase in patients with DVT, SVT, phlebitis, or VI referred to the vascular surgery unit for clinical evaluation.

Nattokinase is an enzyme with thrombolytic and anticoagulant activity [24], documented by Xu et al. [25] in an experimental model of thrombosis_ENREF_18. The thrombolytic effects of nattokinase are suggested by the increase in the fibrin/fibrinogen degradation product D-dimer. Kurosawa et al. [26] reported an increase in D-dimer blood antithrombin concentrations and a decrease in Factor VIII activity with a single nattokinase dose (2000 FU). 

Moreover, it has also been reported that nattokinase induces protection against brain ischemia through its antioxidant and anti-inflammatory activity (i.e., inhibition of nuclear factor-kappa B) [27,28,29]. Moreover, in an experimental study, Ji et al. [10] documented that the neuroprotective effect of nattokinase was associated with its pleiotropic action: (i) anti-platelet activity by elevating cyclic AMP level and attenuating calcium release from calcium stores; (ii) anti-apoptotic effect through the activation of the JAK1/STAT1 pathway; (iii) relaxation of vascular smooth muscle by promoting the synthesis and release of NO, reducing ROC calcium ion influx; (iv) protection on endothelial cells through increasing fibrinolytic activity and facilitating spontaneous thrombolysis.

In previous studies, we documented that several compounds could modulate the expression of matrix metalloproteinases [30,31,32,33,34] involved in endothelial dysfunction. Therefore, nattokinase may also modulate these proteinases, inducing a decrease in inflammation and endothelial diseases.

These findings support our data documenting that nattokinase was able to prevent the development of thrombosis in enrolled patients.

In a previous study, Zhang et al. [35], using a resonance spectroscopy test, documented the interaction between heparin and nattokinase. Recently, co-administration of nattokinase and heparin significantly increased the fibrinolysis activity of nattokinase [36].

It has been reported that soybean fermented with *Bacillus natto* or *Bacillus subtilis* is rich in the ACE inhibitor peptides VAHINVGZK and YVWK [28].

In this regard, Kim et al. [22] randomized 86 patients (20–80 years) with an initial untreated high systolic blood pressure (SBP, from 130 to 159 mmHg) to nattokinase (100 mg/day) or placebo for 8 weeks. The authors documented that nattokinase supplementation decreased systolic blood pressure (5.55 mmHg) and diastolic blood pressure (−2.84 mmHg).

In agreement with this study, Jensen et al. [23] documented that patients treated for 8 weeks with nattokinase (100 mg/day) presented lower plasma levels of von Willebrand factor and platelet factor-4, as well as lower blood pressure compared to their baseline values.

Another surprising observation is that fermented soybean products contain other soy peptides that prevent the onset of type II diabetes [37,38]. In _ENREF_33women (n = 34) with gestational diabetes mellitus (at week 24–28 of gestation), a 6-week consumption of a diet containing soy proteins (35% animal proteins, 35% soy proteins, and 30% other plant proteins) was associated with significant improvements in fasting plasma glucose and serum insulin levels [39]. These data suggest that nattokinase can reduce the risk factors for coronary heart disease and prevent the development of vascular disease. In fact, during the follow-ups, we did not record the development of DVT or DSP.

As reported in international guidelines [40], the first line of treatment of vascular disorders consists in reducing the risk factors by improving both nutrition and lifestyle. Therefore, in this context, nattokinase, a food supplement, can be easily used because it can reduce cardiovascular risk factors.

Finally, another important observation is that we did not record the development of adverse drug reactions, including drug interactions.

These data agree with literature data. In fact, experimental toxicology studies reported the safety of the oral consumption of nattokinase also in humans [41].

However, this study has some limitations, for example, the absence of a placebo control group. In fact, we compared the effect of nattokinase administered alone and as an add-on to anticoagulant compounds but not with respect to an untreated or placebo-treated group; therefore, these data must be validated in a placebo-controlled trial.

## 5. Clinical Risk Management Consideration and Conclusions

Nattokinase (NK) was first presented as a natural drug that can be administered as a mild anticoagulant. It is a natural drug that can be administered both as a single agent and in pharmacological combinations. Although the positive effects and advantages of the drug are greater than the possible described complications, patients under treatment must always be monitored for laboratory parameters to maintain the balance between hyper-coagulation and hypo-coagulation. In fact, patients on a monotherapy with nattokinase or on pharmacological combinations in which one of the drugs prescribed is nattokinase are at risk of thrombus formation or bleeding and hemorrhage. However, to establish the safety of pharmacological treatments of this type, it is necessary to monitor the coagulation parameters and ensure their stability in time, even for drugs known to have mild or no side effects. Coagulation profile, renal function, diet, clinical condition, weight, and age of the patient should be considered as reference parameters. Monitoring implies both constant checks of the laboratory parameters and availability of the results within a few hours from the collection of the blood sample to allow the pharmacological corrections necessary to ensure patient’s safety. In fact, healthy values outside the reference range increase the risk of adverse events. In conclusion, the available evidence suggests that NK is a unique natural compound and represents an effective and safe treatment for patients with vascular disease with medical or surgical indications. The indication of nattokinase could be useful for patients chronically treated with heparin.

## Figures and Tables

**Table 1:** Clinical characteristics of the patients at the time of enrollment. Group 1: patients treated with fondaparinux (7.5 mg/day) and then nattokinase (100 mg/day). Group 2: patients treated with enoxaparin (6000 IU/day) and nattokinase (100 mg/day). Group 3: patients treated with nattokinase (100 mg/day). VAS: visual analogue scale; ESR: erythrosedimentation rate (normal range < 15 mm/h).

Clinical Records	Group 1	Group 2	Group 3
Females	32	34	26
Males	18	23	20
VAS	9	7	7
ESR	22	21	19
Local Skin Redness	+ yes	yes	yes
Local shinny skin	yes	yes	yes
Deep pit edema	Grade 2	Grade 2	Grade 2

## Data Availability

Data are available to the Vascular Surgery Unit of the “Pugliese Ciaccio” Hospital of Catanzaro.
